# Supplementary material for: Submergence deactivates wound-induced plant defence against herbivores
Source: Commun Biol. 2020 Nov 6;3:651. doi: 10.1038/s42003-020-01376-4 (PMC7648080; doi:10.1038/s42003-020-01376-4)
Supplement: Supplementary file 1 — Supplementary Information [file 42003_2020_1376_MOESM1_ESM.pdf]

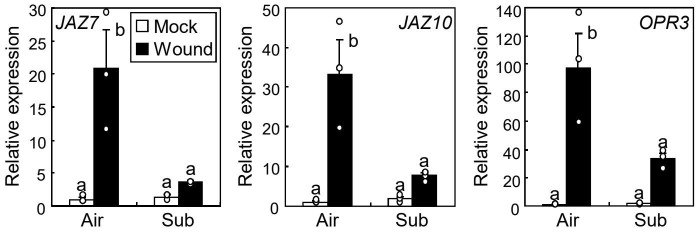

Supplementary Fig. 1 | Wound-induced gene expression after the submergence in soil-grown plants.

Three-week-old Col-0 plants grown in soil were submerged for 1 h and then transferred to the air. Seventh leaf of the reaerated plants were wounded with scissors. The wounded leaves were harvested 1 h after the treatment. Three biological replicates were averaged. Error bars indicate ± s.d. Letters indicate groups that are statistically significantly different from each other ($P < 0.05$, Tukey's test).

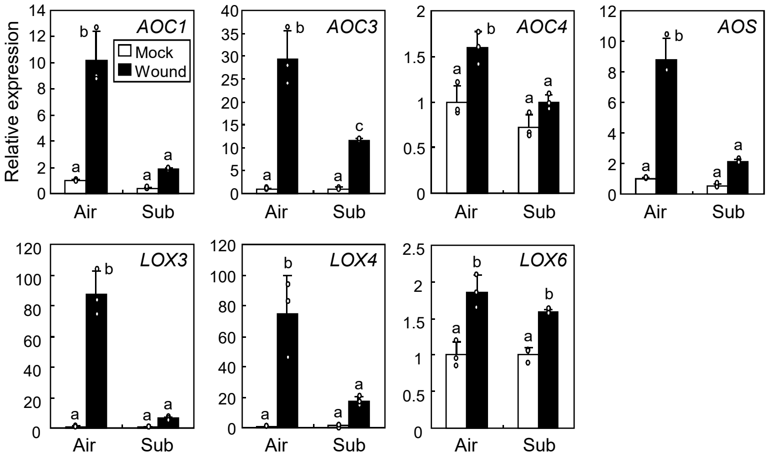

Supplementary Fig. 2 | Expression of JA biosynthesis genes.
Ten-day-old Col-0 seedlings grown on MS-agar plates were used. Seedlings were submerged for 1 h and then transferred to the air. Reaerated plants were wounded immediately. Whole seedlings were harvested 1 h after wounding for total RNA extraction. Three biological replicates were averaged. Error bars indicate ± s.d. Letters indicate groups that are statistically significantly different from each other ($P < 0.05$, Tukey's test).

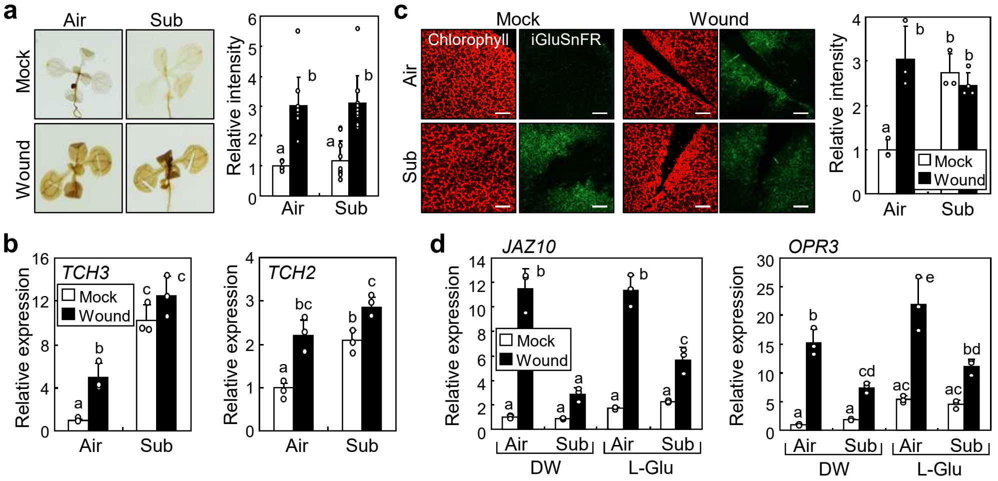

Supplementary Fig. 3 | Early wounding responses after the submergence.
Ten-day-old seedlings grown on MS-agar plates were used. Error bars indicate ± s.d. Letters indicate groups that are statistically significantly different from each other ($P < 0.05$, Tukey's test).
a, H_2O_2 accumulation after the wounding. The Col-0 seedlings were submerged for 1 h and then transferred to the air. Reaerated plants were wounded and subjected to DAB staining 30 min after the treatment. Relative intensities were measured using imageJ software. Six to ten biological replicates were averaged. White and black boxes indicate mock and wound, respectively.
b, Expression of Ca^{2+}-responsive genes after the wounding. The Col-0 seedlings were submerged for 1 h and then transferred to the air. Reaerated plants were wounded immediately and then harvested 1 h after the treatment. Three biological replicates were averaged.
c, Glutamate accumulation after the wounding. The *35S:CHIB-iGluSnFR* transgenic seedlings were submerged for 1 h and then transferred to the air. Reaerated plants were wounded immediately. The iGluSnFR signals and chlorophyll autofluorescence were observed 5 min after the wounding using confocal microscope. Relative intensities were measured using imageJ software. Biological replicates were averaged and statistically analyzed (n = 3~4).
d, Effects of glutamate treatment on wound-responsive genes. The Col-0 seedlings were submerged 1 h and then transferred to the air. Reaerated plants were wounded immediately and then harvested 1 h after the treatment. The 100 mM L-glutamate was sprayed on the seedlings before wounding. Three biological replicates were averaged. Distilled water (DW) was sprayed to the seedlings as a control experiment before submergence.

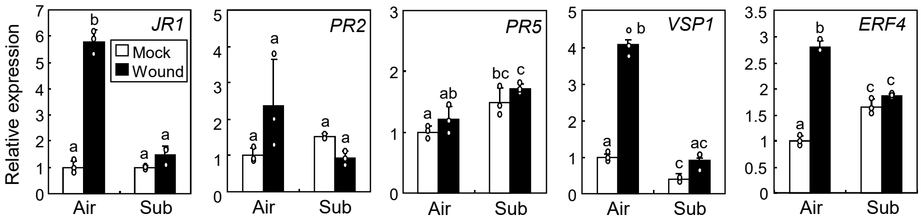

Supplementary Fig. 4 | Expression of JA-responsive genes.
Ten-day-old Col-0 seedlings grown on MS-agar plates were used. Seedlings were submerged for 1 h and then transferred to the air. Reaerated plants were wounded immediately. Whole seedlings were harvested 1 h after wounding for total RNA extraction. Three biological replicates were averaged. Error bars indicate ± s.d. Letters indicate groups that are statistically significantly different from each other ($P < 0.05$, Tukey's test).

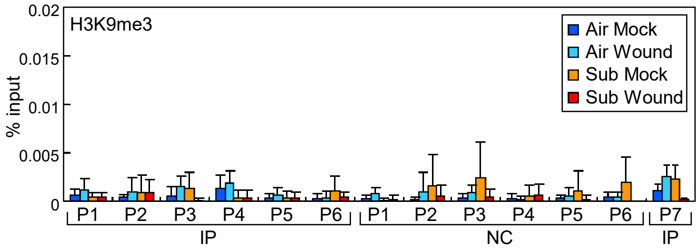

Supplementary Fig. 5 | H3K9me3 levels at *OPR3* locus after the submergence.
Sequence elements used for ChIP assays are annotated as P1 to P7, which are described in Fig. 5a. Ten-day-old Col-0 seedlings grown on MS-agar plates were submerged for 1 h and then transferred to the air. Reaerated plants were wounded immediately. Whole seedlings were harvested for ChIP assays 1 h after the wounding. Anti-H3K9me3 antibody was used for immunoprecipitation. Four biological replicates were averaged. Error bars indicate ± s.d. IP, immunoprecipitation; NC, negative control.

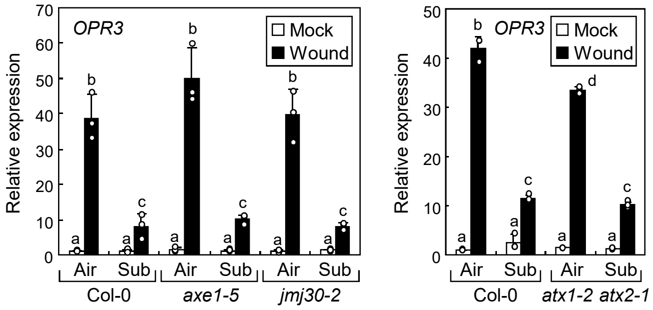

Supplementary Fig. 6 | Expression of the *OPR3* gene in *axe1-5*, *jmj30-2*, and *atx1-2 atx2-1* mutants.
Ten-day-old seedlings grown on MS-agar plates were submerged for 1 h and then transferred to the air. Reaerated seedlings were wounded immediately and harvested 1 h after the treatment. Three biological replicates were averaged. Error bars indicate ± s.d. Letters indicate groups that are statistically significantly different from each other ($P < 0.05$, Tukey's test).

Primers	Sequences	Usage
UBC21-F	CAGTCTGTGTGTAGAGCTATCATAGCAT	RT-qPCR
UBC21-R	AGAAGATTCCCTGAGTCGCAGTT	〃
JAZ7-F	TGAGAAGTTCAGACGGTTCGG	〃
JAZ7-R	TCGAGTCGAATTGTTTGGGAT	〃
JAZ10-F	ATCCCGATTTCTCCGGTCCA	〃
JAZ10-R	ACTTTCTCCTTGCGATGGGAAGA	〃
OPR3-F	TATGGCATGTTGGACGTGCT	〃
OPR3-R	CGGAAGCTTCTAAAGCCCGA	〃
ADH1-F	GAATCGCTGGTGCTTCTAGG	〃
ADH1-R	CTCAGCGATCACCTGTTGAA	〃
AOC2-F	AGGATTCGTTCCTCGCCATC	〃
AOC2-R	GGTGAGCTCCAACGGCAAA	〃
LOX2-F	AGACTGACCAGCGGATTACG	〃
LOX2-R	CCCTTGAGGAACAACTGTCG	〃
TCH2-F	AGCTCAAAGAAGTGATCCG	〃
TCH2-R	TTTCGATTGTTACCTCCTCC	〃
TCH3-F	TACCGTGATGTTTTCCCTCG	〃
TCH3-R	GAGCTCATTCACGGTAATGTAACC	〃
AOC1-F	CAGAACTTGGGAAATACCGA	〃
AOC1-R	AGTTTGTTTGTGAATGGGAC	〃
AOC3-F	CTTAAACTCGGCAAGAAACC	〃
AOC3-R	CTCTTTTCAGGAACGTGTTG	〃
AOC4-F	CAAGAGCTTAACGTCTACGA	〃
AOC4-R	TTCCTATGCGTTTTGTGAGA	〃
AOS-F	CGATTAGCGGAGGAGATTAG	〃
AOS-R	TTCTTCGCTCTACCGTATTG	〃
LOX3-F	TTTGGCTAGACAATCGTTGA	〃
LOX3-R	CCATTCCTCTGCGAATTAGA	〃
LOX4-F	CTTGCTTCTCAAGTTAGGGT	〃
LOX4-R	ATCTCGAAGCCATGCATATT	〃
LOX6-F	AACATGATATGGATCGCCTC	〃
LOX6-R	TATTGAGGGTTTCGCATGAA	〃
ABR1-F	ACAAAGCCAAACTCAACTTC	〃
ABR1-R	GTCTTATGGGCAAAAGGGTA	〃
PDF1.2-F	GCTTTCGACGCACCG	〃
PDF1.2-R	TAGTTGCATGATCCATGTTTG	〃
VSP1-F	CATCTCATACTCAAGCCAAAC	〃
VSP1-R	AGCTTAAAAACCCTTCCAGG	〃
JR1-F	GGACCAGATGAGTATGTCAC	〃
JR1-R	GGTCTTTCTTCTCCAGTACG	〃
PR2-F	TCGAACCAGTGATAGGTTTC	〃
PR2-R	AGTACCCTGGATCGTTATCA	〃
PR5-F	AATGTCAAGCTGGGGATAAG	〃
PR5-R	ATTAAACCTCTCACAGGCAC	〃
ERF4-F	CGTCCTGTCTACTTTTTGGA	〃
ERF4-R	GACCGATGACGAATCAGAG	〃
pJAZ10 BamHI F	ataaatggatccGCGAGCAAACCTTACGCAAA	DNA cloning
pJAZ10 NcoI R	atttatccatggATCAAGACAGAGAGATATGGG	〃
chitinase AscI F	tatggcgcgccAACAATGAAGACTAATCTTTTTCTC	〃
chitinase BamHI R	ataggatccaGGCCGAGGATAATGATAGGAG	〃
iGluSnFR BamHI F	attggatcctaATGGCCGCAGGCAGCACGCTGGAC	〃
iGluSnFR AvrII R	atacctaggTTATTTCAGTGCCTTGTCATTCG	〃
OPR3 P1-F	TGACAAAAATTCGTGACCATC	ChIP-qPCR
OPR3 P1-R	TTATAGAACCTCACTAAGGTGG	〃
OPR3 P2-F	CACAAAAAGAGTTTCCCTGG	〃
OPR3 P2-R	AATGGTTTAGACCAAACTGGA	〃
OPR3 P3-F	CTGACCTGTTAATGGAAGACA	〃
OPR3 P3-R	ATGAGCCTGATTTCCTAAATG	〃
OPR3 P4-F	AGTATTATGCTCAACGGACC	〃
OPR3 P4-R	GTTGACGTCATCTCATCGTA	〃
OPR3 P5-F	ACCGGAGAAAGTTTAAAGGAT	〃
OPR3 P5-R	CTCTATTCTTATCACGCCACT	〃
OPR3 P6-F	AGATGGGATCAATGACCGTA	〃
OPR3 P6-R	CAAGTGATCTATAGCCGGAG	〃
ACT7 P7-F	CGTTTCGCTTTCCTTAGTGTTAGCT	〃
ACT7 P7-R	AGCGAACGGATCTAGAGACTCAC	〃

Supplementary Table 1 | Primers used in this work.
The primers used were designed using the Primer Blast (https://www.ncbi.nlm.nih.gov/tools/primer-blast). F, forward primer; R, reverse primer.
