# Supplementary material for: Submergence deactivates wound-induced plant defence against herbivores
Source: Commun Biol. 2020 Nov 6;3:651. doi: 10.1038/s42003-020-01376-4 (PMC7648080; doi:10.1038/s42003-020-01376-4)
Supplement: Supplementary file 3 — Description of Additional Supplementary Files [file 42003_2020_1376_MOESM3_ESM.pdf]

Description of Additional Supplementary Files

File Name: Supplementary Data 1
Description: Source data to Figure 1.

File Name: Supplementary Data 2
Description: Source data to Figure 2.

File Name: Supplementary Data 3
Description: Source data to Figure 3.

File Name: Supplementary Data 4
Description: Source data to Figure 4.

File Name: Supplementary Data 5
Description: Source data to Figure 5.

File Name: Supplementary Data 6
Description: Source data to Supplementary Figure 1.

File Name: Supplementary Data 7
Description: Source data to Supplementary Figure 2.

File Name: Supplementary Data 8
Description: Source data to Supplementary Figure 3.

File Name: Supplementary Data 9
Description: Source data to Supplementary Figure 4.

File Name: Supplementary Data 10
Description: Source data to Supplementary Figure 5.

File Name: Supplementary Data 11
Description: Source data to Supplementary Figure 6.
